# Comparative metagenomics of biogas-producing microbial communities from production-scale biogas plants operating under wet or dry fermentation conditions

**DOI:** 10.1186/s13068-014-0193-8

**Published:** 2015-02-08

**Authors:** Yvonne Stolze, Martha Zakrzewski, Irena Maus, Felix Eikmeyer, Sebastian Jaenicke, Nils Rottmann, Clemens Siebner, Alfred Pühler, Andreas Schlüter

**Affiliations:** Institute for Genome Research and Systems Biology, CeBiTec, Bielefeld University, Universitätsstraße 25, D-33615 Bielefeld, Germany; QIMR Berghofer Medical Research Institute Herston, 300 Herston Road, Brisbane, QLD 4006 Australia; Bioinformatics Resource Facility, CeBiTec, Bielefeld University, Universitätsstraße 25, D-33615 Bielefeld, Germany; NORTH-TEC Maschinenbau GmbH, Oldenhörn 1, 25821 Bredstedt, Germany

**Keywords:** Biogas, Dry fermentation, Wet fermentation, Microbial communities, Methanogenesis, Metagenomics, 16S rRNA, *Methanoculleus bourgensis*, Fragment recruitment

## Abstract

**Background:**

Decomposition of biomass for biogas production can be practiced under wet and dry fermentation conditions. In contrast to the dry fermentation technology, wet fermentation is characterized by a high liquid content and a relatively low total solid content. In this study, the composition and functional potential of a biogas-producing microbial community in an agricultural biogas reactor operating under wet fermentation conditions was analyzed by a metagenomic approach applying 454-pyrosequencing. The obtained metagenomic dataset and corresponding 16S rRNA gene amplicon sequences were compared to the previously sequenced comparable metagenome from a dry fermentation process, meeting explicitly identical boundary conditions regarding sample and community DNA preparation, sequencing technology, processing of sequence reads and data analyses by bioinformatics tools.

**Results:**

High-throughput metagenome sequencing of community DNA from the wet fermentation process applying the pyrosequencing approach resulted in 1,532,780 reads, with an average read length of 397 bp, accounting for approximately 594 million bases of sequence information in total. Taxonomic comparison of the communities from wet and dry fermentation revealed similar microbial profiles with *Bacteria* being the predominant superkingdom, while the superkingdom *Archaea* was less abundant. In both biogas plants, the bacterial phyla *Firmicutes*, *Bacteroidetes*, *Spirochaetes* and *Proteobacteria* were identified with descending frequencies. Within the archaeal superkingdom, the phylum *Euryarchaeota* was most abundant with the dominant class *Methanomicrobia*. Functional profiles of the communities revealed that environmental gene tags representing methanogenesis enzymes were present in both biogas plants in comparable frequencies. 16S rRNA gene amplicon high-throughput sequencing disclosed differences in the sub-communities comprising methanogenic *Archaea* between both processes. Fragment recruitments of metagenomic reads to the reference genome of the archaeon *Methanoculleus bourgensis* MS2^T^ revealed that dominant methanogens within the dry fermentation process were highly related to the reference.

**Conclusions:**

Although process parameters, substrates and technology differ between the wet and dry biogas fermentations analyzed in this study, community profiles are very similar at least at higher taxonomic ranks, illustrating that core community taxa perform key functions in biomass decomposition and methane synthesis. Regarding methanogenesis, *Archaea* highly related to the type strain *M. bourgensis* MS2^T^ dominate the dry fermentation process, suggesting the adaptation of members belonging to this species to specific fermentation process parameters.

**Electronic supplementary material:**

The online version of this article (doi:10.1186/s13068-014-0193-8) contains supplementary material, which is available to authorized users.

## Background

Rising energy costs and considerations on long term environmental sustainability have placed renewable energy sources in the focus of debate. The development of renewable energy resources offers the chance to replace traditional fossil fuels and can help to reduce carbon dioxide emissions [1,2]. An economically attractive technology to generate bioenergy is the production of biogas that is a mixture of methane (CH_4_) and carbon dioxide (CO_2_) as the main components, with small amounts of hydrogen sulfide (H_2_S), nitrogen (N_2_), hydrogen (H_2_), ammonia (NH_3_) and carbon monoxide (CO) [3]. The most common and widespread utilization of biogas is the production of electricity and heat by its combustion in combined heat and power units.

The process of biogas production takes place under anaerobic conditions and involves microbial decomposition of organic matter, yielding methane as the main final product of underlying metabolic pathways. In Germany, mostly maize silage combined with liquid manure is utilized as the substrate for biogas production [1,4]. Complex consortia of microorganisms are responsible for biomass decomposition and biogas production involving the stages substrate hydrolysis, acidogenesis, acetogenesis and methanogenesis. However, most of these microbes, as well as their roles in biogas production, are currently unknown. Recently, the analysis of the structure, composition and activity of microbial communities in relation to input substrates and fermentation parameters in biogas plants have become the focus of research [5-7]. It is generally accepted that a better understanding of the composition and activity of the multifarious microbial community is crucial for further optimization of reactor performance and fermentation process technologies. Moreover, to increase the yield of biogas, a detailed insight into relevant microbial metabolic pathways involved in methane synthesis and syntrophy is necessary.

Previous studies analyzed the taxonomic structure and enzymatic potential of biogas communities residing in agricultural biogas reactors. A classical microbiological approach for the analysis of the communities’ taxonomy is the generation of 16S rRNA gene clone libraries, followed by Sanger sequencing of the 16S rRNA gene fragments [8-12]. Sequencing of 16S rRNA gene clone libraries is limited since coverage of the microbial complexity frequently is laborious, costly and time-consuming. Moreover, sequence information on community 16S rRNA marker genes does not provide direct insights into functions of microorganisms. To achieve deeper insights into community structure and function, metagenome analyses applying high-throughput sequencing technologies were carried out [13-17]. Elaborate bioinformatics methods and analysis platforms facilitated metagenome sequence data interpretation and comparison [13,14,16,18]. Another approach for comparison of metagenome datasets is fragment recruitment of metagenomic sequences related to selected genomes of reference species. This approach provides insights into the degree of relatedness of indigenous species within a given habitat to known reference species. Recently, fragment recruitment has been applied for marine and silage microbial communities [19,20].

In principle, decomposition of biomass for biogas production can be practiced under wet or dry fermentation conditions. Wet fermentation is characterized by a high liquid content and a low total solid content, which usually is below 10%. In contrast to this, in dry fermentation the total solids content is between 15 and 35%. Biogas plants operating under dry fermentation conditions apply mostly maize silage, green rye (and similar biomass), dung (cow dung, poultry dry excrement and so forth) or municipal solid wastes as substrates without any continuous supplementation of liquid manure, which consequently leads to a low liquid content [1,21]. To control the water content, recirculation of digestate or liquid is applied, which may have a great impact on the activity of the underlying community. Recirculation may influence the pH, salt and organic loads, which could inhibit the microbial activity. Dry fermentation proved to be a convenient technology for the fermentation of substrates possessing relatively high dry matter contents. According to the German Renewable Energy Law (EEG), a technology bonus was granted for dry fermentation biogas plants built before the year 2008. Previously, a dry fermentation process of a production-scale biogas plant was characterized at the metagenomic level [13,14,16]. On the other hand, wet fermentation utilizing maize silage and liquid manure from cattle or swine is performed in most mid-sized, agricultural biogas plants in Germany. In this study, the microbial community of an agricultural biogas plant performing wet fermentation was analyzed by applying a metagenomic approach. Obtained results were compared to taxonomic community profiles deduced from a dry fermentation biogas plant analyzed previously. The present study adopted exactly the same methodology for processing samples, preparing total community DNA and metagenome sequence data analysis as for metagenome analysis of the dry fermentation process in the study mentioned above. It is hypothesized that biogas-producing microbial communities comprise a ‘core’ microbiome and variable sub-communities that respond to specific conditions and process parameters prevailing in particular reactor environments. The objective of this study was to differentiate biogas communities from biogas plants performing wet and dry fermentation, with respect to their taxonomic profiles, and to deduce correlations between these profiles and process parameters collected for both fermentation types. Another aim of this study was to identify key species specifically adapted to one process or the other and their predicted functions, focusing on methanogenic species.

## Results and discussion

### Analyzed biogas production plants differ in substrate input and chemical parameters

To compare taxonomic and functional profiles of the biogas-producing microbial communities from production-scale biogas plants operating under dry or wet fermentation conditions, samples from the primary digesters of two agricultural biogas plants differing in these fermentation types were analyzed. The biogas plant operating under dry fermentation conditions (BGP_DF) was sampled previously [16], whereas samples from the biogas plant operating under wet fermentation conditions (BGP_WF) were taken in March 2011. One of the major differences between BGP_WF and BGP_DF is their dry matter content (BGP_DF: 14% ± 2% and BGP_WF: 9% ± 1%). BGP_DF was fed with high amounts of plant silages and low amounts of chicken manure [16], whereas the substrate composition of BGP_WF mainly consisted of maize silage and a relatively high amount of liquid pig manure (Table 1). Moreover, both biogas plants can be distinguished according to their process parameters, such as volatile organic acids, total inorganic carbon, acetic and propionic acid concentrations and ammonium contents (see Table 1). BGP_DF is characterized by higher acetic and propionic acid concentrations as compared to BGP_WF, suggesting that consumption of these compounds is limited in BGP_DF. The biogas and methane yields (698.2 l/kg and 350.5 l/kg organic dry matter (oDM), see Table 1) are in the normal range of production in mesophilic production-scale biogas plants.

**Table 1:** **Characteristics of the studied biogas plants performing wet or dry fermentation technology**

	**Biogas plant operating dry fermentation (Sampling date: 14 August 2007)**	**Biogas plant operating wet fermentation (Sampling date: 1 March 2011)**
**pH**	7.7 ± 0.01	7.8 ± 0.01
**Conductivity (mS/cm)**	17.1 ± 1	21.6 ± 1
**VOA (mg/l)**	7,739 ± 60	3,987 ± 31
**TIC**	15,159 ± 120	14,517 ± 115
**VOA/TIC**	0.51	0.27
**NH**_**4**_ **-N (g NH**_**4**_ **-N/l)**	2.25 ± 0.02	2.85 ± 0.02
**Acetic acid (mg/l)**	2,628 ± 50	344 ± 7
**Propionic acid (mg/l)**	179 ± 3.6	14 ± 0.3
**Fed substrates**	Maize silage (63%), green rye (35%), chicken manure (2%)	Maize silage (approximately 72%), pig manure (approximately 28%)
**Biogas yield (l/kg oDM)**	698.2	810.5
**Methane yield (l/kg oDM)**	350.5	417.8

oDM: Organic dry matter; TIC: Total inorganic carbon; VOA: Volatile organic acids.

In a recent study, a metagenome approach was carried out to study the taxonomic composition and functional potential of the microbial community in the biogas plant BGP_DF [13,16]. In total, 1,347,644 sequencing reads were generated with an average read length of 367.7 bases providing approximately 496 million bases sequence information (Table 2). Most biogas plants in Germany practice wet fermentation utilizing liquid manure and maize silage for the production of methane. To obtain insights into the microbial community composition of this process, a metagenome sequencing approach for the biogas plant applying wet fermentation (BGP_WF) was carried out. The same sample preparation, DNA-extraction method and sequencing technique were applied as previously described for BGP_DF to ensure comparability of the metagenome datasets. Sequencing of the samples originating from BGP_WF resulted in 1,532,780 sequencing reads, with an average read length of 387.3 bases, accounting for approximately 594 million bases sequence information (Table 2). To include only high quality sequences, both datasets were filtered for GC (G: Guanine, C: Cytosine bias and duplicates as previously described [22]. After this filtering step, 1,019,333 sequences from the BGP_DF and 1,097,549 sequences from the BGP_WF remained and were used for downstream taxonomic and functional analyses (see Table 2).

**Table 2:** **Metagenome sequencing statistics of the DNA samples from the wet (BGP_WF) and dry (BGP_DF) fermentation biogas plants**

	**Unfiltered sequences** ^**a**^		**Filtered sequences** ^**a**^	
	**BGP_DF**	**BGP_WF**	**BGP_DF**	**BGP_WF**
**Reads (bp)**	1,347,644	1,532,780	1,019,333	1,097,549
**Average read length (bp)**	368	387	366.0	387.5
**Sequence information (Mbp)**	495.5	593.7	373.1	424.3

^a^Sequencing data summary is shown before (unfiltered sequences) and after (filtered sequences) the filtering step for duplicates and GC bias. Mbp: mega base pairs.

### Comparative analyses of taxonomic profiles obtained from wet and dry fermentation communities revealed high similarities

The community structures in the biogas plants operating under wet (BGP_WF) or dry fermentation (BGP_DF) conditions were studied using CARMA3 [23] and MetaSAMS [24]. The software CARMA3 was applied to calculate the taxonomic (microbial composition based on phylogenetic analyses) and functional profile (predicted Pfam protein families based on similarity searches) of the microbial community. CARMA3 is implemented in MetaSAMS (Metagenome Sequence Analysis and Management System), a software suited for the analysis of metagenome datasets. For the interpretation only taxonomic assignments with an E-value threshold of 10^−5^ were considered. In total, 711,293 sequences of the BGP_WF were assigned to a superkingdom representing 64.8% of the total number of analyzed sequences. In the BGP_DF dataset, 604,243 sequences (59.3% of the total sequences) were similar to known reference sequences at the rank superkingdom. Rarefaction analyses on the mean taxonomic richness on the taxonomic family rank showed a saturation from approximately 800,000 reads, which indicates that the majority of the microbial community has been captured, while the rarefaction analysis on the rank genus was nearly saturated (Additional file 1: Figure S1A and D).

**Figure 1:**
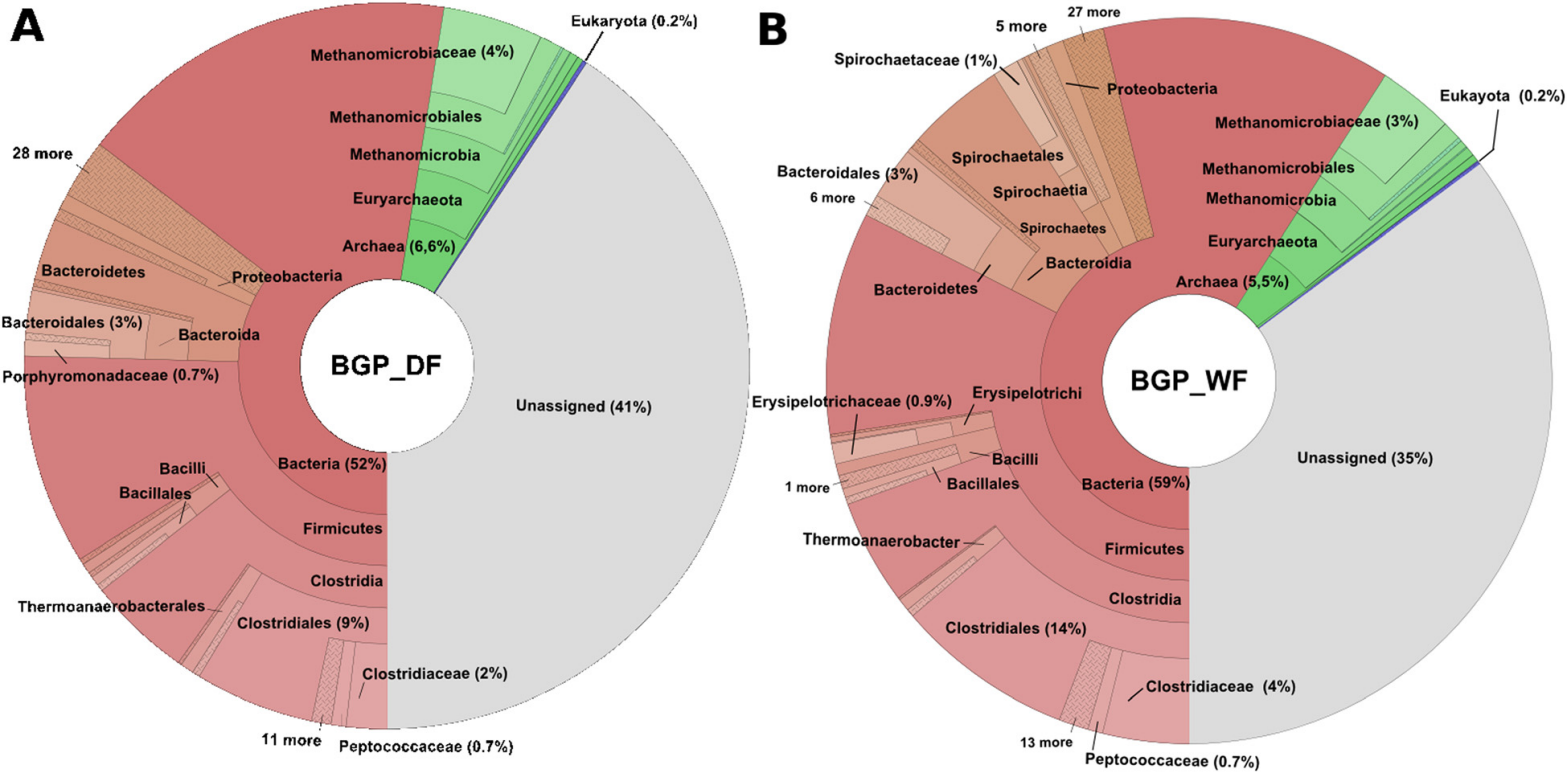
**Taxonomic composition of microbial communities from the wet and dry fermentation processes.** Taxonomic structure of the microbial community associated with a **(A)** dry (BGP_DF) or **(B)** wet (BGP_WF) fermentation process: The taxonomic composition of the microbial communities residing in the biogas plants analyzed was determined using CARMA3 and visualized by Krona plots. Only the ranks from superkingdom to family were considered.

At higher taxonomic ranks, the community compositions in BGP_WF and BGP_DF are very similar (Figure 1). Both communities are mainly composed of bacterial (59% in BGP_WF and 52.5% in BGP_DF of the total analyzed reads) and archaeal microorganisms (5.5% in BGP_WF and 6.6% in BGP_DF) (Figure 1). The bacterial superkingdom mainly comprises the phyla *Firmicutes* (32.5% in BGP_WF and 25.4% in BGP_DF), *Bacteroidetes* (8.4% in BGP_WF and 6.2% in BGP_DF), *Spirochaetes* (1.7% in BGP_WF and 0.5% in BGP_DF) and *Proteobacteria* (1.7% in BGP_WF and 1.2% in BGP_DF). These phyla were also common in other biogas plant microbiota [9,17,18]. Bacterial groups belonging to the taxa *Firmicutes*, *Bacteroidetes* and *Spirochaetes* are assumed to be involved in cellulolytic degradation, proteolysis, acidogenesis and homoacetogenesis [10]. Among the archaeal community, *Euryarchaeota* (5.2% in BGP_WF and 6.2% in BGP_DF of all analyzed sequences) is the most abundant phylum with *Methanomicrobia* as the largest class (Figure 1). Comparing both profiles on the phylum level, minor differences were observed in the relative abundances of *Firmicutes*. However, there are some noteworthy changes in the relative abundances of taxonomic groups between wet and dry fermentation digesters on the class level, in particular of classes belonging to the phylum *Firmicutes*.

Among the phylum *Firmicutes* in BGP_WF, *Clostridia* (19.5% of all analyzed sequences in BGP_WF) forms the largest class, followed by *Bacilli* (1.8%) and *Erysipelotrichi* (1.2%) (Figure 1). Likewise, *Clostridia* and *Bacilli* are also the most abundant classes of *Firmicutes* in the microbiome of the BGP_DF, with 14.3% and 1.4% of the total number of analyzed reads, respectively, whereas *Erysipelotrichi* is barely present (0.1%) in this digester. While *Clostridia* and *Bacilli* species are well described in the anaerobic digestion process in biogas plants, the evidence for *Erysipelotrichi* species is sparse [248]. Little is known about the family *Erysipelotrichaceae.* However, it was also identified in the microbial community of the gut [25]. An increase in members of this family was associated with a diet high in fat, increased body weight and decreased fecal short-chain fatty acid concentrations in mice [25]. Concurrently, occurrence of *Erysipelotrichi* members correlates with lower short-chain fatty acid concentrations in BGP_WF. Whether this observation is really due to the metabolic capabilities of this group of microorganisms remains to be determined.

**Figure 2:**
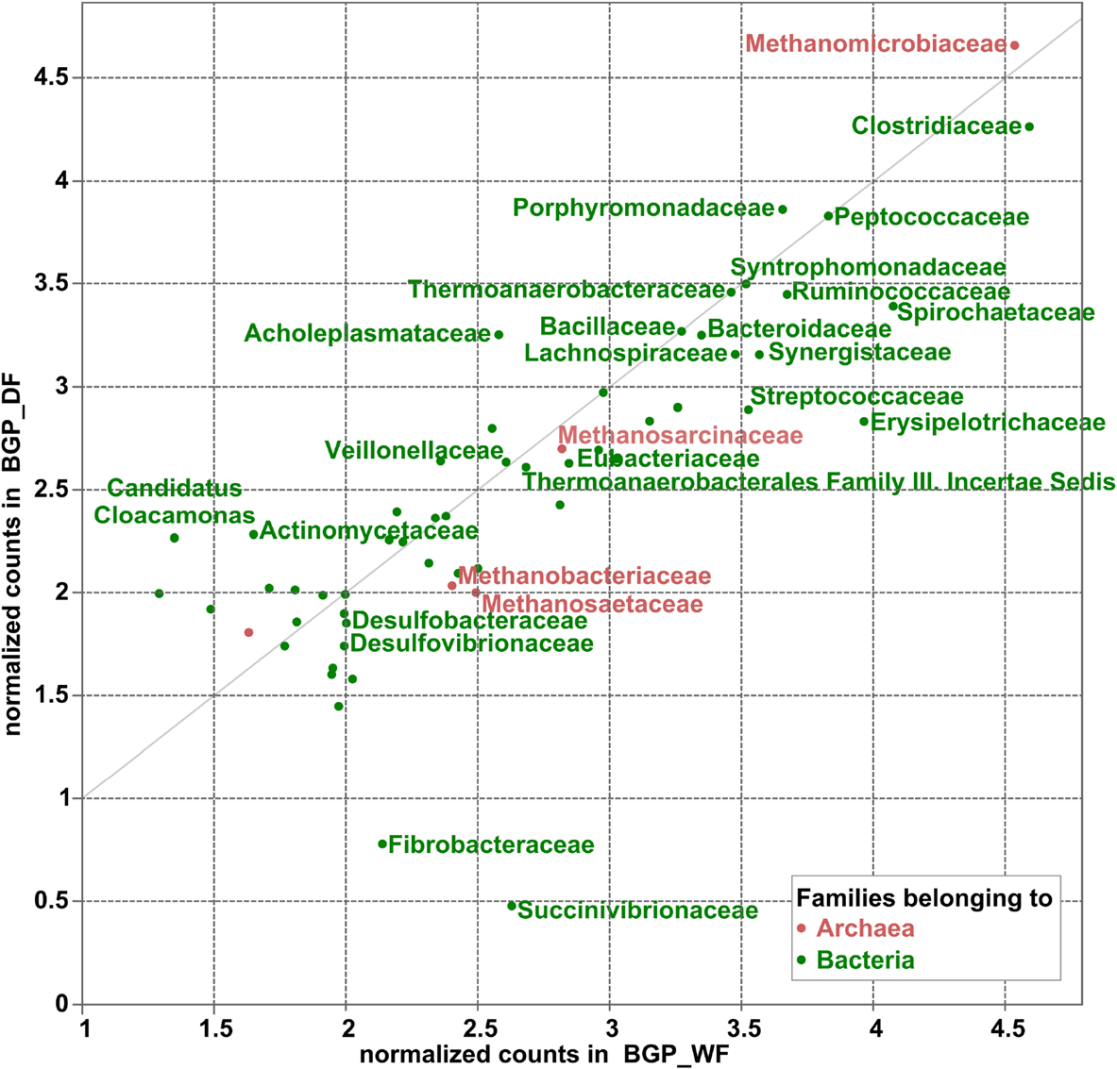
**Scatterplot of sequence counts assigned to the taxonomic rank family for microbial communities from the wet and dry fermentation processes.** For each taxonomic family, sequence abundances, normalized on the smallest dataset, with pseudocounts (for an explanation see Methods section) are plotted as a logarithm to the base 10. Red symbols indicate archaeal and green symbols indicate bacterial families. Only families for which the sum of the relative read abundances measured was at least 0.01% were considered.

In both biogas plants, the most abundant families belonging to the class *Clostridia* are *Clostridiaceae* (3.8% in BGP_WF and 1.8% in BGP_DF of all analyzed sequences), *Ruminococcaceae* (0.5% in BGP_WF and 0.3% in BGP_DF) and *Lachnospiraceae* (0.3% in BGP_WF and 0.1% in BGP_DF) (Figures 1 and 2). *Clostridium*, the prevalent genus within *Clostridiaceae*, seems to belong to the core set of organisms, as it is dominant in both biogas plants studied (Figure 1). Species of this genus, such as *Clostridium thermocellum* [26] and *Clostridium clariflavum* [27], produce cellulosomes, an extracellular multi-enzyme complex which is important for the degradation of complex carbohydrates such as cellulose. Indeed, environmental gene tags (EGTs) classified to the genus *Clostridium* encode enzymes relevant in the hydrolysis process of glycoside bonds (PF00150 and PF00759). In both biogas plants, *Alkaliphilus* is the second largest genus within the family *Clostridiaceae.* It has also been detected in high amounts in a biogas plant fed with plant biomass and pig manure slurry [17]. The species *Alkaliphilus peptidofermentans*, isolated from a soda lake, is described to ferment peptides to acetate and formate [28]. The functional profile of EGTs assigned to the genus *Alkaliphilus* in the wet fermentation process includes various peptidase families (PF00768 and PF05343).

Members of the genus *Ruminococcus* belonging to the family *Ruminococcaceae* (order *Clostridiales*) are cellulolytic organisms, which occur in the rumen community [29] and in biogas plants [17]. They play an important role in the digestion of plant cell wall material and produce acetate [29,30]. Among the functional profile of EGTs assigned to *Ruminococcus*, protein families for glycosyl hydrolase family 9, 26 and 48 (PF00759, PF02156 and PF04616) representing cellulose-, cellobiose- and hemicellulose-degrading enzymes were observed. Furthermore, gene fragments for dockerin and cohesin (PF00404 and PF00963), the scaffoldin units of cellulosomes, were detected in the functional profile of the wet fermentation process. Recently, the genome of *Acetivibrio cellulolyticus* of the family *Ruminococcus* was sequenced [31]. The genome carries genes for a complex cellulosome system including endoglucanases and cellobiohydrolases. Glycosyl family 8 and 9, both representing these enzymes, were detected among the EGTs assigned to the genus *Acetivibrio*.

The family *Spirochaetaceae* dominates the phylum *Spirochaetes* with 68% of the reads assigned to this phylum in BGP_DF (Figure 1). It is the third largest family, with 1.2% of all reads in the wet fermentation sample, whereas it belongs to the minor groups in the dry fermentation process (0.2%). Microorganisms related to *Treponema* have already been described in a mesophilic biogas digester treating pig manure using 16S rRNA clone libraries [10]. Genomes of the *Treponema* species encode proteolytic enzymes [32] and glycoside hydrolases [33]. EGTs classified to the genus *Treponema* in BGP_WF were also assigned to carbohydrate phosphorylase (PF00343), alpha amylase (PF00128) and 4-alpha-glucanotransferase (PF02446), which participate in starch and sucrose metabolism, carbohydrate phosphorylase (PF00343) and alphaamylase (PF00128). The family *Succinivibrionaceae* belonging to the *Gammaproteobacteria* is only predicted in the biogas plant operating wet fermentation (0.04% of all analyzed sequenced reads) and was sparsely detected in the dry fermentation process (Figures 1 and 2). This family is noted to use glucose and other carbohydrates as an energy source and to produce succinate and acetate [34]. Likewise, the family *Fibrobacteraceae* is more frequently present in the wet fermentation process. The known species *Fibrobacter succinogenes* is described to play a key role in the degradation process of cellulose [35].

*Prevotellaceae* belonging to the phylum *Bacteroidetes* is more abundant in the BGP_WF, with 0.18% of all analyzed sequences in the corresponding dataset as compared to 0.8% of all sequences in the BGP_DF (Figures 1 and 2). Species of the genus *Prevotella* are described to decompose hemicellulose, starch and pectin [36,37]. Various Pfam families were discovered in the wet fermentation digester that were assigned to *Prevotella* and are predicted to be involved in the degradation of hemicellulose (such as glycosyl hydrolase family 3 (PF00933, PF01915), glycosyl hydrolase 92 (PF07971) and alpha-L-arabinofuranosidase (PF06964)) or starch (such as glycosyl hydrolase family 31 (PF01055)).

*Synergistaceae* of the phylum *Synergistetes* belongs to the core-set of the families represented in both biogas plants (0.4% in BGP_WF and 0.1% in BGP_DF of all analyzed reads) (Figures 1 and 2). *Anaerobaculum* and *Aminobacterium* are genera within the phylum *Synergistetes*, which are both predicted in the biogas plants. Recently, *Anaerobaculum* was detected in anaerobic digestion of slaughterhouse waste mixture [38] and thermophilic sludge [11]. Species of *Anaerobaculum* are associated with the fermentation of peptides and produce short-chain fatty acids. Likewise, species of the genus *Aminobacterium* were isolated from anaerobic sludge and are described to ferment a range of amino acids to acetate, propionate and hydrogen [39,40].

### Community profiles of the dry and wet fermentation process differ at lower taxonomic ranks

There are also a number of taxa that are slightly increased in the biogas plant operating the dry fermentation technology (Figures 1 and 2). For example, the two biogas plants differ in the proportion of sequences belonging to the family *Acholeplasmataceae*, of the phylum *Tenericutes*. The species *Acholeplasma laidlawii* was isolated from wastewater [15] and also has been identified in other biogas plants [1]. As a source for carbon, *A. laidlawii* utilizes glucose, fructose and galactose [15]. The genome of *A. laidlawii* harbors genes for enzymes that degrade starch, amino sugars and other sugars. In the functional profile of EGTs assigned to this family are glycoproteases (PF00814) and peptidases (PF01546) (data not shown).

In the dry fermentation process, *Candidatus Cloacamonas* is more prevalently present (Figure 2)*.* The species *Candidatus Cloacamonas acidaminovorans* was recently detected to be highly abundant in other anaerobic digesters [17,41]. Previously, the genus was also identified in a 16S rRNA clone library of the same biogas plant [42]. Proteome analysis indicated that *C. acidaminovorans* might attain energy from sugars in the Embden-Meyerhof pathway and from the fermentation of amino acids, and thereby produces hydrogen and carbon dioxide [41].

Overall, the taxonomic profiles are similar for the wet and dry anaerobic digestion, especially at higher taxonomic ranks. In the BGP_WF, the families *Erysipelotrichaceae*, *Fibrobacteraceae*, *Succinivibrionaceae* and *Clostridiaceae* were found to be more abundant, whereas more sequences were assigned to *Acholeplasmataceae* and *Candidatus Cloacamonas* in the BGP_DF.

### Hydrogenotrophic methanogens are dominant in the dry and wet fermentation processes

A detailed taxonomic analysis was performed for the archaeal sequences of both fermentation processes. In the wet fermentation process, less EGTs were assigned to *Methanomicrobiaceae* (3.4%) as compared to the dry fermentation digester (4.4%) (Figures 1 and 2). In both fermentation processes, *Methanomicrobiaceae* is the most abundant methanogenic family with *Methanoculleus* being the prevalent genus. *Methanoculleus* species conduct the hydrogenotrophic methanogenesis pathway, synthesizing methane from carbon dioxide and hydrogen. Further identified families that are capable of performing methanogenesis are *Methanosarcinaceae, Methanosaetaceae* and *Methanobacteriaceae* [43]*.* These families are only detected in low abundance in the microbial communities. In the BGP_WF, the acetoclastic methanogen *Methanosaeta* was measured in slightly higher frequencies as compared to the dry fermentation process (Figure 2). This genus was noted to be more dominant in biogas plants with low acetate concentrations [44], as it has a high affinity to acetate. The observation is supported by this study as the abundance of *Methanosaeta* correlates with the lower concentration of acetic acid in BGP_WF compared to BGP_DF (see Table 1).

**Figure 3:**
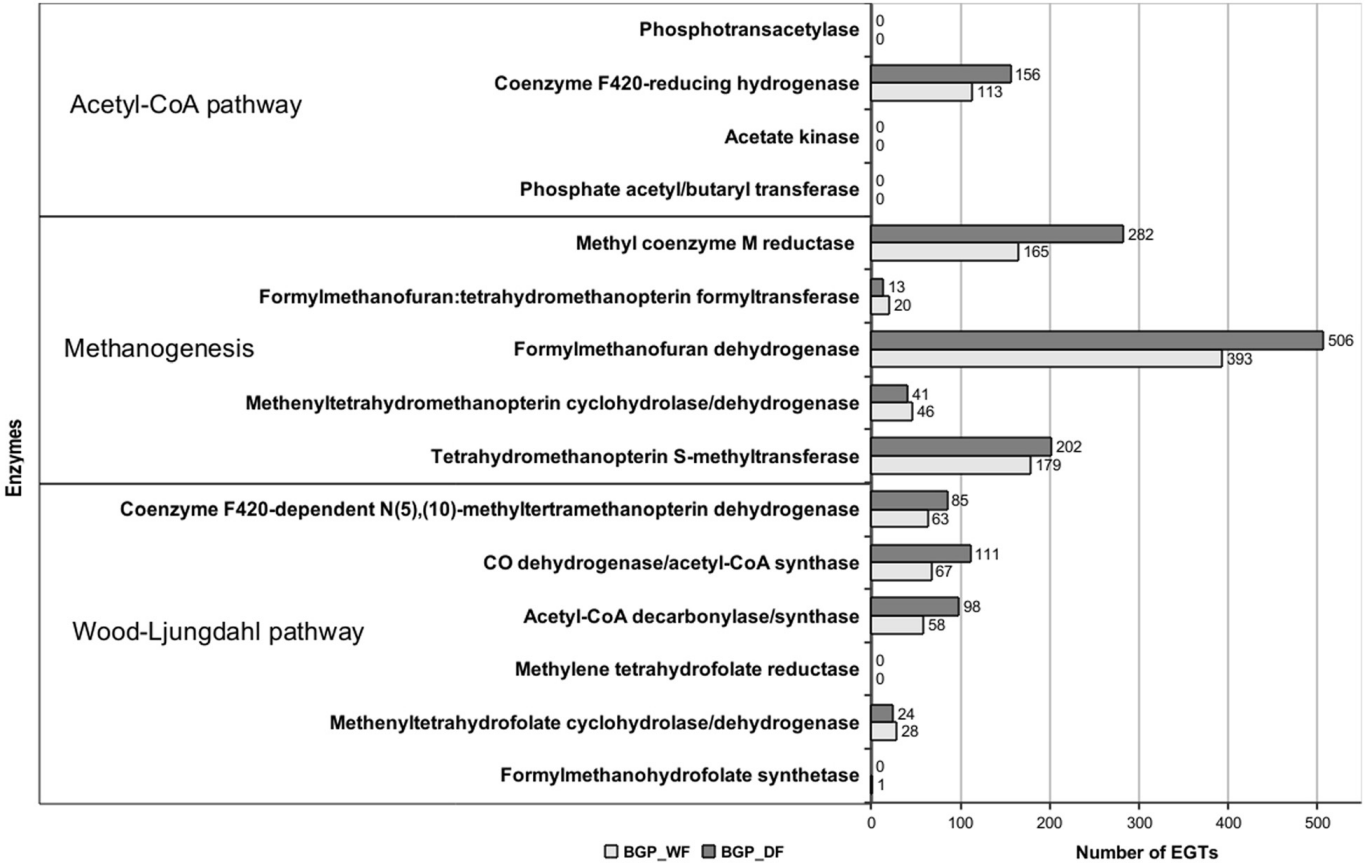
**Comparative analysis of archaeal environmental gene tags (EGTs) representing methanogenesis-related enzymes in metagenome datasets for the wet or dry fermentation process.** Comparison of the relative abundances of EGTs representing enzymes involved in the acetyl-CoA pathway, methanogenesis and Wood-Ljungdahl pathway. Only EGTs assigned to *Archaea* were considered and relative abundances were normalized based on the smallest dataset.

In addition to the taxonomic comparison of the biogas plants, enzymes involved in the acetoclastic and hydrogenotrophic methanogenesis were categorized according to Pfam families and were searched in the functionally characterized metagenomes obtained from BGP_WF and BGP_DF. Reads assigned to the selected Pfam families and classified to the superkingdom *Archaea* were used for the subsequent analysis (see Figure 3). No EGTs were assigned to acetate kinase and phosphotransacetylase, which are key enzymes in the initial step of acetoclastic methanogenesis in *Methanosarcina* [45]. However, EGTs for all enzymes relevant in the hydrogenotrophic methanogenesis pathway were identified with a slightly higher amount in the dry fermenter (data not shown). Rarefaction analyses on the mean EGT richness showed a saturation at approximately 800,000 reads, which indicates that the majority of the microbial community has been captured (see Additional file 1: Figure S1B). Moreover, we emphasized the EGTs that are unique in each of the biogas plants’ metagenomes, which means that they are absent in the one plant while they have an abundance of at least five in the other plant (normalized on the smallest dataset). This analysis showed that there are more unique EGTs in the wet fermentation process metagenome. However, none of the identified unique EGTs can be correlated to functions directly associated with the fermentation processes leading to methane production (Additional file 2: Table S1).

### Differentiation of the methanogenic sub-community within the wet and dry fermentation process

To analyze the archaeal sub-community at a higher resolution, high-throughput 16S rRNA gene amplicon sequencing was carried out. The sequencing procedure for BGP_WF yielded 83,719 sequencing reads. A previous rRNA gene amplicon sequencing run of the sample obtained from BGP_DF resulted in 170,941 sequencing reads [46]. In total, 37 operational taxonomic units (OTUs) were of archaeal origin, representing 2,753 sequences of the BGP_WF dataset (8%). After manual inspection for chimeric sequences and excluding those sequences with a length below 100 bp, 15 OTUs remained comprising 2,691 reads. In the dry fermentation process, 22 OTUs representing 1,118 sequences (2%) were assigned to *Archaea*. After removing manually detected chimeric and short (<100 bp) sequences, nine OTUs remained representing 1,095 sequences. The number of sequences included in the OTUs was normalized to the number of the smallest filtered 16S rRNA gene amplicon dataset. Phylogenetic analysis of representative OTU sequences that were assigned to the superkingdom *Archaea* was carried out using FastTree. Rarefaction analyses on the mean OTU richness showed saturation at approximately 30,000 reads, which indicates that the majority of the microbial community has been captured (see Additional file 1: Figure S1C).

**Figure 4:**
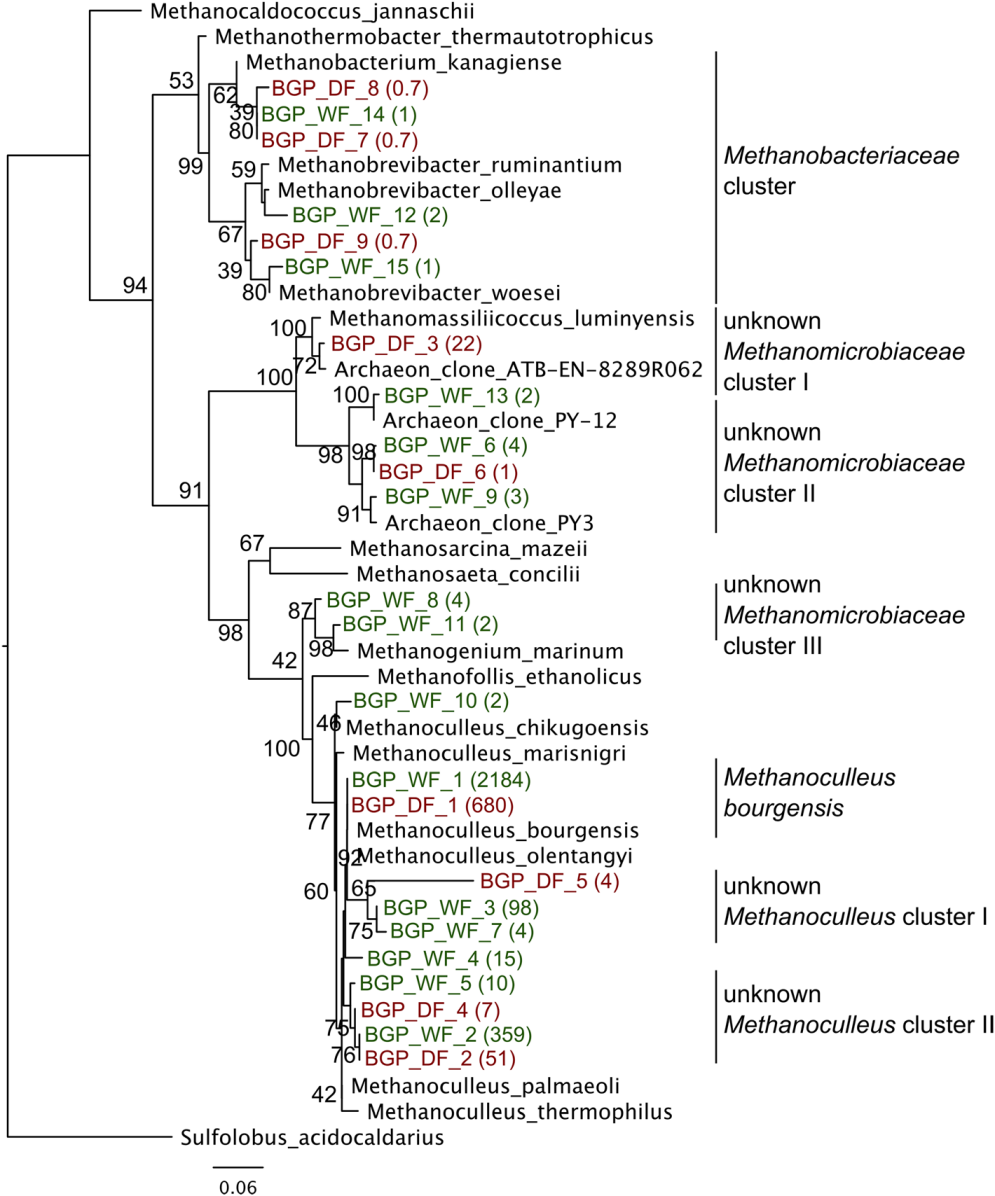
**Archaeal phylogenetic tree of representative operational taxonomic units (OTUs) sequences from the wet and dry fermentation processes.** Representative OTU sequences and corresponding counts assigned from the wet (BGP_WF, red) or dry fermentation (BGP_DF, green) process are labeled with an OTU identifier in brackets, and the nodes are labeled with the respective boot strap values. Representative sequences were assigned to one known Methanobacteriaceae and three unknown Methanomicrobiaceae clusters (I to III), and to one known and two unknown *Methanoculleus bourgensis* clusters (I and II), respectively. Sequence counts were normalized according to the smallest dataset.

The largest archaeal OTUs of the wet fermentation and dry fermentation plant are similar to *Methanoculleus bourgensis* with 2,184 and 680 sequences, respectively (Figure 4). The presence of a phylogenetic cluster related *to M. bourgensis* is in agreement with previous studies based on 16S rRNA clone library analyses obtained from the biogas plant BGP_DF [42]. The representative sequences of the second largest OTU of each biogas plant cluster together. The representative amplicon sequences comprise 359 and 51, respectively, and are located in a large cluster formed by known *Methanoculleus* species. However, no sequence of a described *Methanoculleus* species could be identified in close proximity of these representative sequences, indicating a so far unknown archaeal species related to *Methanoculleus* (unknown *Methanoculleus* cluster II).

A further unknown *Methanoculleus* species is highly represented in the wet fermentation plant (unknown *Methanoculleus* cluster I). The corresponding OTU contains 98 sequence reads. A representative sequence of the dry fermentation biogas plant clusters close to the latter OTU, comprising only four reads. A so far unknown *Methanoculleus* species has also been described in the same biogas plant based on 16S rRNA library clones [42].

Moreover, representative sequences are located outside the phylogenetic cluster formed by the described *Methanoculleus* species. Corresponding archaeal species also belong to the class *Methanomicrobia* (cluster I and cluster II). The phylogenetic tree distantly affiliated a sequence from the dry fermentation plant to the recently identified species *Methanomassiliicoccus luminyensis* B10 from a human gut microbiome sample [47]. The representative sequence also clusters close to a so far unknown archaeal clone [48], which originates from an agricultural biogas plant supplied with water, maize silage and barley grains [49]. The distribution of similar sequences in various habitats suggests a wide occurrence of species related to *M. luminyensis*.

Besides *Methanomicrobia*, *Methanobacteria* were identified in the biogas plants based on phylogenetic analysis. Corresponding species are related to *Methanobrevibacter* and *Methanobacterium*. Both were identified in the 16S rRNA gene amplicon dataset, as well as in the whole metagenome approach, in the two biogas plants with minor frequencies. Some representative sequences (BGP_DF_8, BGP_WF_7 and BGP_WF_14) form a phylogenetic cluster with the 16S rRNA gene sequence of *Methanobacterium kanagiense* [50], a hydrogenotrophic archaeon isolated from an anaerobic propionate-oxidizing soil sample. Another representative sequence (BGP_WF_12) is 96% identical to the 16S rRNA gene sequence of *Methanobrevibacter olleyae* [51]. This species is present in sheep and bovine rumen and was described to use hydrogen and formate for methane formation. Further representative sequences (BGP_DF_9 and BGP_WF_15) are associated with *Methanobrevibacter woesei* and feature an identity of 97 to 98% to this species. *M. woesei* was isolated from goose gut and is a hydrogenotrophic archaeon that mainly uses hydrogen and carbon dioxide for methane production.

Finally, no representative sequences related to acetoclastic methanogens were identified in the biogas plants, showing a dominance of hydrogenotrophic methanogens in the biogas production process of these plants.

Overall, the constructed archaeal phylogenetic tree illustrates differences in the composition of the methanogenic sub-communities of both biogas plants. Besides the dominating *M. bourgensis* further *Methanoculleus* species are also present in the dry fermentation process (unknown *Methanoculleus* cluster I and II). In BGP_WF other methanogens comprising species related to unknown *Methanomicrobiaceae* species (cluster II and cluster III (see Figure 4) are prominent.

### Fragment recruitments revealed that methanogens of the dry fermentation plant are closely related to the type strain *Methanoculleus bourgensis* MS2^T^ at the genomic level

To analyze the degree of relatedness of biogas-producing community members to completely sequenced reference microorganisms, and to differentiate the metagenome datasets of both biogas plants referring to this, fragment recruitments were conducted as described previously [19]. Another objective of this analysis was to determine similarities of methanogens of both biogas plants to the genome of the type strain *M. bourgensis* MS2^T^ [43]. For this purpose, metagenome sequence reads were searched for matches to completely sequenced microbial genomes stored in the National Center for Biotechnology Information (NCBI) database applying the BLASTn algorithm [Basic Local Alignment Search Tool on nucleotide level]. Counts of metagenome reads that match to reference genomes with more than 90% sequence similarity are listed for the top 20 genomes in Table 3. Reference organisms appearing in this analysis taxonomically represent the orders *Methanomicrobiales*, *Clostridiales*, *Lactobacillales*, *Thermoanaerobacterales* and *Synergistales* and could be classified to belong to one of the following functional groups: cellulolytic organisms, secondary fermenters (acidogenic), syntropic organisms (acetogenic) and methanogens (see Table 4). Also, for BGP_DF, the orders *Thermotogales* and *Bacillales* were identified among the top 20 recruitments.

**Table 3:** **List of 20 reference genomes showing the highest similarities to the metagenome datasets from biogas plants operating under wet or dry fermentation conditions as analyzed by fragment recruitment analysis**

**Reference sequence**	**Wet fermentation** ^**a**^	**Dry fermentation** ^**a**^
*Methanoculleus bourgensis* MS2^T^	15,992 (1.19%)	59,969 (4.45%)
*Clostridium clariflavum* DSM 19732	3,840 (0.28%)	3,282 (0.24%)
*Clostridium thermocellum* ATCC 27405	2,464 (0.18%)	2,201 (0.16%)
*Clostridium kluyveri* DSM 555	1,423 (0.11%)	807 (0.06%)
*Streptococcus infantarius* subsp. infantarius CJ18	1,360 (0.1%)	367 (0.03%)
*Thermoanaerobacterium thermosaccharolyticum* DSM 571	1,234 (0.09%)	1,454 (0.11%)
*Mahella australiensis* 50–1 BON	1,179 (0.09%)	1,319 (0.1%)
*Methanoculleus marisnigri* JR1	1,014 (0.08%)	3,298 (0.24%)
*Desulfotomaculum carboxydivorans* CO-1-SRB	944 (0.07%)	682 (0.05%)
*Clostridium difficile* M120	776 (0.06%)	386 (0.03%)
*Thermoanaerovibrio acidaminovorans* DSM 6589	688 (0.05%)	387 (0.03%)
*Syntrophomonas wolfei* subsp. wolfei str. Goettingen	622 (0.05%)	358 (0.03%)
*Streptococcus gallolyticus* UCN34	621 (0.05%)	191 (0.01%)
*Streptococcus suis* GZ1	575 (0.04%)	444 (0.06%)
*Streptococcus pasteurianus* ATCC 43144	556 (0.04%)	176 (0.01%)
*Streptococcus macedonicus* ACA-DC 198	523 (0.04%)	153 (0.01%)
*Thermoanaerobacter* sp. X514	502 (0.04%)	310 (0.02%)
*Pelotomaculum thermopropionicum* SI	452 (0.03%)	533 (0.04%)
*Clostridium cellulolyticum* H10	437 (0.03%)	321 (0.02%)
*Clostridium cellulovorans* 743B	386 (0.03%)	210 (0.01%)

^a^Number and percentage of reads recruited for each strain determined by BLASTn analyses (reads featuring at least 90% sequence similarity to the reference genome were counted).

**Table 4:** **Species identified during a fragment recruitment analysis using metagenome sequence data of wet and dry fermentation biogas plants**

**(x)** ^**a**^ **Species (y)** ^**b**^	**Functional role**	**Taxonomy** ^**c**^ **(phylum, class, order)**	**Origin; attributes; metabolic features**	**Reference**
(1) *Methanoculleus bourgensis* (1)	*Methanogenic*	*Euryarchaeota, Methanomicrobia, Methanomicrobiales*	Isolated from activated sludge; methanogen; hydrogenotroph	[43]
(2) *Clostridium clariflavum* (3)	*Cellulolytic*	*Firmicutes, Clostridia, Clostridiales*	Isolated from thermophilic anaerobic sludge; Cluster III *Clostridium*; cellulolytic; cellulosome	[52]
(3) *Clostridium thermocellum* (4)	*Cellulolytic*	*Firmicutes, Clostridia, Clostridiales*	Isolated from hot spring (Yellowstone), cotton bales, farm soil and other habitats; thermophilic; cellulolytic; cellulosome	[26,53]
(4) *Clostridium kluyveri* (7)	*Secondary fermenters*	*Firmicutes, Clostridia, Clostridiales*	Isolated from canal mud; fermentation of ethanol and acetate to butyrate, caproate and H_2_	[54]
(5) *Streptococcus infantarius* (13)	*Secondary fermenters*	*Firmicutes, Bacilli, Lactobacillales*	Isolated from fermented dairy and plant products; associated with different human and animal infections; fermentative metabolism	[27]
(6) *Thermoanaerobacterium thermosaccharolyticum* (5)	*Syntrophic*	*Firmicutes, Clostridia, Thermoanaerobacterales*	Isolated from geothermal sites (Yellowstone); class V *Clostridia*; saccharolytic; fermentation of a wide range of carbohydrates to ethanol, acetic acid, lactic acid, H_2_ and CO_2_	[55,56]
(7) *Mahella australiensis* (6)	*Secondary fermenters*	*Firmicutes, Clostridia, Thermoanaerobacterales*	Isolated from oil field (Queensland, Australia); predicted to utilize pentoses; xylose metabolism	[57]
(8) *Methanoculleus marisnigri* (2)	*Methanogenic*	*Euryarchaeota, Methanomicrobia, Methanomicrobiales*	Isolated from marine sediment; methanogen; hydrogenotroph	[58,59]
(9) *Desulfotomaculum carboxydivorans* (8)	*Secondary fermenters*	*Firmicutes, Clostridia, Clostridiales*	Isolated from anaerobic bioreactor sludge; moderately thermophilic; fermentation of pyruvate, lactate, glucose and fructose; chemolithoheterotrophic; sulfate reduction	[60]
(10) *Costridium difficile* (12)	*Secondary fermenters*	*Firmicutes, Clostridia, Clostridiales*	Human isolate; pathogenic for humans and animals; causes diarrhea and colitis; mesophilic; chemoorganotroph	[61]
(11) *Thermoanaerovibrio acidaminovorans* (11)	*Syntrophic*	*Synergistetes, Synergistia, Synergistales*	Isolated from anaerobic reactor of a sugar refinery; fermentation of amino acids to acetate and propionate; metabolism enhanced by hydrogen scavenger	[62]
(12) *Syntrophomonas wolfei* subsp. wolfei (15)	*Syntrophic*	*Firmicutes, Clostridia, Clostridiales*	Isolated from anaerobic digester sludge; syntrophic fatty acid metabolism, syntrophic association with methanogenic archaeon	[63]
(13) *Streptococcus gallolyticus* (-)	*Secondary fermenters*	*Firmicutes, Bacilli, Lactobacillales*	Isolated from endocarditis patient; part of the rumen flora; pathogenic for ruminants, birds and humans; fermentation of carbohydrates of plant origin	[18,64-67]
(14) *Streptococcus suis* (10)	*Secondary fermenters*	*Firmicutes, Bacilli, Lactobacillales*	Clinical origin; zoonotic pathogen for pigs and humans; fermentation of carbohydrates	[2,32,67]
(15) *Streptococcus pasteurianus* (-)	*Secondary fermenters*	*Firmicutes, Bacilli, Lactobacillales*	Isolated from human blood; pathogenic; fermentation of carbohydrates	[52,67]
(16) *Streptococcus macedonicus* (-)	*Secondary fermenters*	*Firmicutes, Bacilli, Lactobacillales*	Isolated from fermented (dairy) foods; pathogenic; fermentation of carbohydrates	[67,68]
(17) *Thermoanaerobacter* sp*.* (18)	*Secondary fermenters*	*Firmicutes, Clostridia, Thermoanaerobacterales*	Isolated from deep sub-surface sample; thermophilic; fermentation of monomeric and polymeric carbohydrates to ethanol	[69,70]
(18) *Pelotomaculum thermopropionicum* (9)	*Syntrophic*	*Firmicutes, Clostridia, Clostridiales*	Isolated from granular sludge of an upflow blanket reactor; thermophilic; fermentation of volatile fatty acids (propionate) in syntrophic association with methanogen	[71,72]
(19) *Clostridium cellulolyticum* (16)	*Cellulolytic*	*Firmicutes, Clostridia, Clostridiales*	Isolated from decayed grass compost; cellulolytic; cellulosome	[55]
(20) *Clostridium cellulovorans*	*Cellulolytic*	*Firmicutes, Clostridia, Clostridiales*	Isolated from methanogenic fermentation of hybrid poplar wood; mesophilic; cellulolytic; cellulosome	[73]
(-) *Petrotoga mobilis* (14)	*Secondary fermenters*	*Thermotogae, Thermotogae, Thermotogales*	Isolated from hot oil-field water from oil reservoir; heterotrophic; fermentation of different carbohydrates including xylan	[74]
(-) *Bacillus coagulans* (17)	*Secondary fermenters*	*Firmicutes, Bacilli, Bacillales*	Isolated from spoiled canned milk; thermotolerant; slightly acidophilic; carbohydrate utilization; production of lactic acid	[75]
(-) *Geobacillus* sp. (19)	*Secondary fermenters*	*Firmicutes, Bacilli, Bacillales*	thermophilic; chemoorganotrophic	CP001638 (GenBank Accession No., unpublished)
(-) *Syntrophothermus lipocaldicus* (20)	*Syntrophic*	*Firmicutes, Clostridia, Clostridiales*	Isolated from thermophilic upflow anaerobic sludge blanket; utilization of fatty acids (butyrate); syntrophic association with hydrogenotrophic organisms	[76]

^a^Ranking of fragment recruitments within the BGP_WF dataset.

^b^Ranking of fragment recruitments within the BGP_DF dataset.

^c^Taxonomic classification of the reference microorganism identified by fragment recruitment. Identified microorganisms were classified according to their functional role.

**Figure 5:**
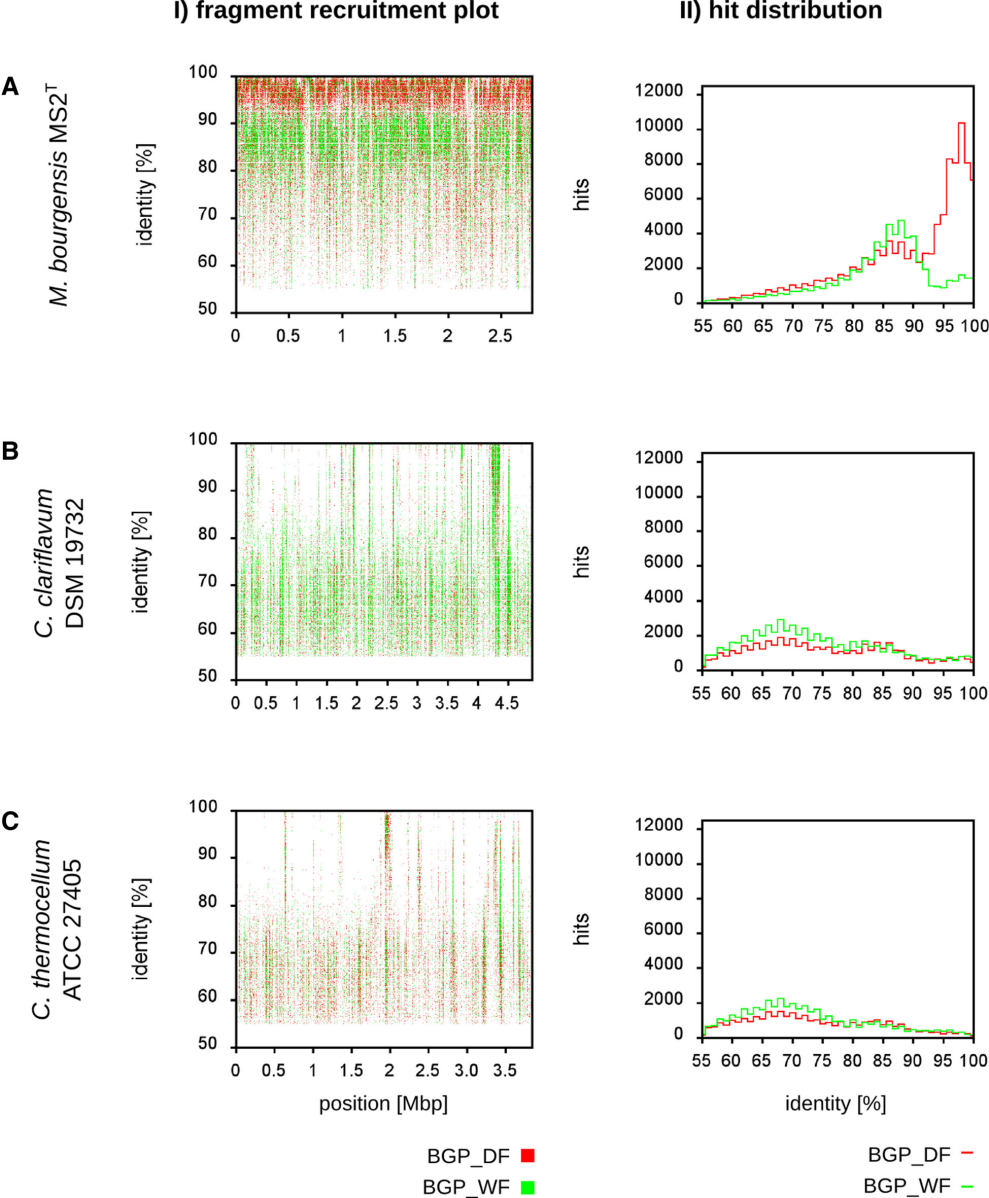
**Fragment recruitment plots and hit distribution for metagenome sequence reads from the wet and dry fermentation processes on three reference genomes.** Visualization of BLASTn analyses of metagenomic sequence reads to the reference genomes of *Methanoculleus bourgensis* MS2^T^ **(A)**, *Clostridium clariflavum* DSM 19732 **(B)** and *Clostridium thermocellum* ATCC 27405 **(C)**. Within the fragment recruitment plot (I) the sequence identities (>55%) between each hit of a metagenomic sequence read and the chromosomal reference sequence are plotted against the position of the alignment. In the hit distribution plot (II) the normalized numbers of reads featuring hits to the reference genome were plotted in intervals of 1% for metagenome reads showing 55 to 100% sequence identity to the reference.

Additionally, BLASTn results were graphically evaluated in fragment recruitment plots, in which the degree of similarity of a single hit is plotted against the position of this hit on the reference genome sequence. This was done for those three microorganisms showing the highest sequence similarities to matching metagenome sequence reads (see Table 3), namely *M. bourgensis* MS2^T^, *C. clariflavum* DSM 19732 and *C. thermocellum* ATCC 27505 (see Figure 5). Hit distribution plots show the numbers of hits with a similar degree of similarity to the reference genome sequence. Fragment recruitments revealed that methanogens within fermentation samples of both biogas plants are related to the reference type strain *M. bourgensis* MS2^T^. It was observed that dominant methanogens of BGP_DF are more closely related to the reference genome as compared to those of BGP_WF (see Figure 5A). It was supposed that *M. bourgensis* may be very well adapted to adverse conditions prevailing in BGP_DF, such as high ammonium concentrations and osmolarity [12,77,78], due to the fed substrate, which was chicken dry excrement.

Surprisingly, *M. bourgensis* MS2^T^ represents the only organism that can be regarded as reasonable reference for members of the biogas-producing communities analyzed in this study. All other microorganisms listed in Table 3 feature a lesser degree of conservation to biogas community members and hence are more distantly related to them (exemplarily see Figure 5B,C). Moreover, differentiation of the microbial communities of both biogas plants is not apparent from these fragment recruitment analyses, since results obtained for both metagenome datasets uncovered nearly the same set of prominent reference organisms, with only slight differences in rankings (see Tables 3 and 4). This result confirms deduced taxonomic profiles for both biogas plants at higher taxonomic ranks which did not uncover pronounced differences between both microbial communities (see previous sections). However, it was observed that the diversity within the genus *Streptococcus* is greater for BGP_WF which was fed with swine manure. It is known that particular *Streptococcus* species such as *Streptococcus suis* and *Streptococcus pasteurianus* belong to the common microbiome of the pig’s intestinal tract [52,79,80]. Moreover, it should be noted here that *Desulfotomaculum carboxydivorans* was identified at rank nine for BGP_WF and at rank eight for BGP_DF in fragment recruitments. This bacterium is able to catabolize low-molecular weight compounds (pyruvate, lactate, glucose etc.) and reduces sulfate as terminal electron acceptor to sulfide (hydrogen sulfide), which is deleterious in biogas since it leads to corrosion of combustion units and in pipelines.

In summary, fragment recruitments revealed that representation of biogas community members in databases is currently insufficient. The only exception is *M. bourgensis* MS2^T^ which is very similar to dominant members of the methanogenic sub-community, at least in BGP_DF.

## Conclusions

The methane-producing community of an agricultural biogas plant applying wet fermentation technology was characterized by means of a metagenomic approach and taxonomically compared with the microbial community of a biogas plant applying dry fermentation that was previously characterized [16]. This is the first study in which metagenomes of different biogas plants are compared using the same DNA preparation technique, sequencing technology and bioinformatics methods. Both communities’ metagenomes were sequenced using 454-pyrosequencing, and the sequence data were analyzed based on their taxonomic composition and functional profile, with focus on the methanogenic sub-community and genes involved in methanogenesis, using the same bioinformatics tools and pipelines. Despite differences in the process parameters, such as acetic acid concentration and pH value and the biogas plant’s substrate compositions, the microbial communities of BGP_WF and BGP_DF are similar in their composition on higher taxonomic ranks. Only a minor number of taxa on lower ranks differed between BGP_WF and BGP_DF. Accordingly, the majority of the taxa belonged to the core set of microorganisms residing in both biogas plants. Even though in both fermenters, the family of *Methanomicrobiaceae* is most dominant and *Methanoculleus* is the prevalent genus within this family, the composition of this genus differs between the different plants. As fragment recruitments revealed, within the genus *Methanoculleus*, *M. bourgensis* was the most dominant species in dry fermentation while this was not the case for the wet fermentation process.

The presented results clearly indicate that the hypothesis of a core community being present in biogas fermenters, operating either under wet or dry fermentation conditions, can be confirmed. This is in accordance with several studies examining the microbial taxonomic composition in biogas fermenters. Identified differences in taxonomic profiles can be associated with different process parameters of the fermenters, such as fed substrate, pH, acid and ammonium concentrations, as especially methanogens are sensitive regarding changes in acetate and propionate concentrations.

To further verify the hypothesis of a core microbiome and an adaptation of specific species to particular process parameters, further comparative studies with more biogas fermenters are required. Analyzing microbiota in biogas plants with altered physiochemical characteristics (temperature, pH and concentration of relevant metabolites) and substrate supply would aid in identifying the core species essential for the anaerobic digestion process, which in turn would provide information to control the biogas production process and prevent unstable conditions. Moreover, the metatranscriptome of the microbial biogas communities should be studied and compared to corresponding metagenomes to enable characterization not only of the taxonomic composition, but also to deduce the actual metabolic activity within the biogas fermenters.

## Methods

### Total community DNA isolation, purification and sequencing

The biogas plant featuring the mesophilic continuous dry fermentation technology (hereafter noted as BGP_DF) was designed for a capacity of 530 kW_el_ (combined heat and power (CHP)) and a daily input of maize silage (63%), green rye (35%) and chicken manure (2%), divided into 24 feedings per day. The process comprises two digesters; the primary digester (BIOGAS NORD GmbH, Bielefeld, Germany) (1,557 m^3^_net volume_, height of 6 m, diameter of 19 m) has an organic load of 4.8 kg oDM m^−3^ d^−1^, a theoretical retention time of 59 days and a temperature of 40°C. At the end, the digestate is stored in a closed non-heated final storage reactor (BIOGAS NORD GmbH, Bielefeld, Germany) (2,987 m^3^_net volume_, height of 6 m, diameter of 26 m). The biogas and methane yields at the time of sampling were at 698.2 and 350.5 l/kg oDM, respectively.

The biogas plant applying the mesophilic wet fermentation technology (hereafter BGP_WF) was designed for a capacity of 537 kW_el_ CHP. The daily input of maize silage (approximately 72%) and liquid pig manure (approximately 28%) was divided into 24 feedings per day. The biogas plant is composed of two digesters and the storage tank (BIOGAS NORD GmbH, Bielefeld, Germany). The digester (2,041 m^3^_net volume_, height of 6.4 m, diameter of 21 m) has an organic load of 4.0 kg oDM m^−3^ d^−1^, a theoretical retention time of 55 days and a temperature of 40°C. The digestate is stored in a closed non-heated final storage tank (4,742 m^3^_net volume_, height of 6 m, diameter of 32 m). The biogas and methane yields at the time of sampling were at 810.5 and 417.8 l/kg oDM, respectively.

Samples were taken from the primary digester of BGP_WF and total community DNA was extracted in triplicates applying the same procedure as described previously for the biogas plant featuring dry fermentation (BGP_DF). The triplicates were pooled prior to sequencing applying high-throughput sequencing using the Genome Sequencer FLX system (Roche Diagnostics GmbH, Mannheim, Germany) [22]. The BGP_WF metagenomic DNA was sequenced omitting the additive for high-GC DNA in order to ensure comparability between the metagenomes of the BGP_DF and BGP_WF. Subsequently, both sequence datasets were processed to remove emulsion-PCR duplicates and sequences affected by GC-biases in the course of sequencing, as described previously [22].

### Taxonomic and functional analysis of metagenome sequence data

The filtered metagenome sequences of BGP_DF and BGP_WF were imported into MetaSAMS [81] and taxonomically characterized using CARMA3 [23]. CARMA3 computes taxonomic and functional assignments for EGTs derived from a microbial community. The filtered metagenome sequences of BGP_DF and BGP_WF were imported into MetaSAMS to apply CARMA3, which is implemented in MetaSAMS. Moreover, MetaSAMS enables exploration, analysis, management and visualization of calculated observations for metagenome sequences. Using MetaSAMS, rarefaction curves were calculated based on the levels ‘family’ and ‘genus’. Rarefaction analysis addresses the assessment of ‘taxon’ richness from different sub-sample sizes regarding metagenome sequence reads. In a rarefaction curve, the number of assigned taxa (on a specified level) is plotted as a function of the number of sequences within a selected sub-sample. The functional profile was calculated by searching for Pfam families matching with an E-value threshold of 10^−5^. Subsequently, the functional profile for selected taxonomic units was evaluated regarding their functional roles in the anaerobic digestion process.

To determine whether the relative abundance of specific taxa changed in the biogas fermenters, abundances were visualized in a scatter plot. First, the absolute counts of reads assigned to a specific family were normalized according to the smallest dataset. Then, the logarithm was used to decrease the influence of more dominant families. To allow the logarithm of zero, a pseudo-count of one sequence was added for each family prior to the logarithm. Moreover, differences in the methanogenesis step were studied. Briefly, EGTs which were taxonomically characterized as originating from archaeal species and functionally assigned to genes involved in the acetyl-CoA, methanogenesis or Wood-Ljungdahl pathway were counted and normalized according to the smallest sample [19]. The metagenomic sequence data of BGP_WF can be found at the European Molecular Biology Laboratory - European Bioinformatics Institute (EMBL-EBI) database under the accession number [EMBL: PRJEB5813].

### 16S rDNA amplicon generation, sequencing and analysis

16S rRNA gene amplicons were generated and sequenced as described recently [24], applying high-throughput sequencing using the Genome Sequencer FLX system (Roche Diagnostics GmbH, Mannheim, Germany). First, a PCR was performed to amplify a region covering V3 and V4 using universal 16S rDNA primers. Next, gel electrophoresis and gel extraction were applied to obtain only amplicons with the correct length. The PCR was repeated in order to attach barcode tags as well as adaptors to the amplicons. Finally, the amplicons were sequenced on a 454 Genome Sequencer (GS) device using FLX Titanium chemistry.

16S rRNA amplicon sequences of the BGP_DF and BGP_WF were simultaneously processed using the QIIME (Quantitative Insights Into Microbial Ecology) pipeline [82]. First, barcode and primer sequences were removed allowing 0 and 2 mismatches, respectively, and sequences with ambiguous bases were discarded. The option ‘truncate_only’ was used meaning that reverse primer sequences will be trimmed only if they are identified at the end of the amplicon sequences. To obtain high quality sequences for phylogenetic analyses, strict quality processing was carried out (window size of 25 bases, average quality score 25). Subsequently, the software package USEARCH version 6.0 was applied for denoising, chimera detection (*de novo* mode) and clustering into OTUs based on a 97% sequence identity [83,84]. Afterwards, representative sequences were selected for each cluster and assigned to taxonomic groups using the RDP Classifier 2.5 [85]. Only assignments with a confidence value of at least 0.8 were considered. Rare-faction curves based on OTUs were calculated to determine the coverage of the microbial community by the sequenced metagenome reads. For phylogenetic analysis, representative sequences assigned to *Archaea* with a confidence value of at least 0.8 were selected and aligned with the Infernal 1.1 software [86] using the *Archaea* SSU rRNA model (RF01959) from Rfam [48]. Finally, the alignment was used as a basis for tree reconstruction using FastTree [87]. The tree was rooted with the sequence of the *Crenarchaeota Sulfolobus acidocaldarius* covering the V3-V4 region.

### Fragment recruitments

Fragment recruitments were performed as described previously [19,20] to compare the relatedness of metagenomic reads to the genomes of reference microorganisms. BLASTn analyses of metagenomic reads against a database containing all genomes of completely sequenced microorganisms were accomplished. Identity values of hits were computed by dividing the number of identical bases by the sequence length of the read. Hits with an identity value below 55% were discarded. Results of the BLASTn analysis were then visualized by plotting the calculated identity of each sequence read against the alignment position on the reference sequence. Moreover, a histogram was generated to display the distribution of hit identities. Numbers of hits displayed in fragment recruitments and in histograms were normalized based on the smallest sample size.

## Additional files

Additional file 1: Figure S1. Rarefaction analyses of sequenced metagenomes and 16S rRNA gene amplicons originating from dry (BGP_DF) and wet fermentation biogas plants (BGP_WF) microbial communities. Rarefaction analysis plots on (A) taxonomic mean richness at the family rank derived from metagenome data, (B) environmental gene tags (EGT) derived from metagenome data and (C) operational taxonomic unit (OTU) richness derived from 16S rRNA gene amplicons in correlation with the sampled reads. Additional file 2: Table S1. Unique environmental gene tags (EGTs) encoding different proteins found in metagenome datasets for the wet and dry fermentation processes. Only EGTs featuring an abundance of 0 in the one and at least five in the other metagenome dataset were taken into account. Relative abundances were normalized based on the smallest dataset.

## Abbreviations

BGP_DF: Biogas plant operating under dry fermentation conditions
BGP_WF: Biogas plant operating under wet fermentation conditions
EGT: Environmental gene tag
oDM: Organic dry matter
OTU: Operational taxonomic unit
