# Supplementary figures and images for: Comparative metagenomics of biogas-producing microbial communities from production-scale biogas plants operating under wet or dry fermentation conditions
Source: Biotechnol Biofuels. 2015 Feb 8;8:14. doi: 10.1186/s13068-014-0193-8 (PMC4329661; doi:10.1186/s13068-014-0193-8)

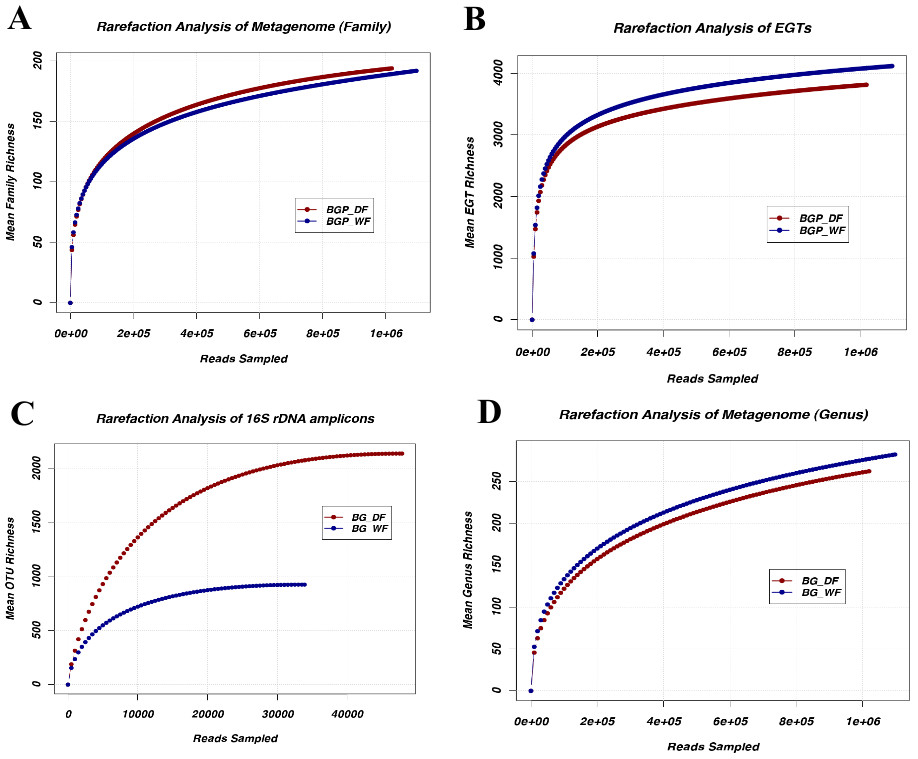

Supplement: Additional file 1: Figure S1. — Rarefaction analyses of sequenced metagenomes and 16S rRNA gene amplicons originating from dry (BGP_DF) and wet fermentation biogas plants (BGP_WF) microbial communities. Rarefaction analysis plots on (A) taxonomic mean richness at the family rank derived from metagenome data, (B) environmental gene tags (EGT) derived from metagenome data and (C) operational taxonomic unit (OTU) richness derived from 16S rRNA gene amplicons in correlation with the sampled reads. [file 13068_2014_193_MOESM1_ESM.png]
