# Supplementary material for: Comparative metagenomics of biogas-producing microbial communities from production-scale biogas plants operating under wet or dry fermentation conditions
Source: Biotechnol Biofuels. 2015 Feb 8;8:14. doi: 10.1186/s13068-014-0193-8 (PMC4329661; doi:10.1186/s13068-014-0193-8)
Supplement: Additional file 2: Table S1. — Unique environmental gene tags (EGTs) encoding different proteins found in metagenome datasets for the wet and dry fermentation processes. Only EGTs featuring an abundance of 0 in the one and at least five in the other metagenome dataset were taken into account. Relative abundances were normalized based on the smallest dataset. [file 13068_2014_193_MOESM2_ESM.doc]

**SUPPLEMENTARY TABLE**

Table 1. **Unique** **environmental gene tags (EGTs) encoding different proteins found in metagenome datasets for the wet or dry fermentation process**

| **Unique genes found in the dry fementation metagenome (> 5 EGTs)** | **Unique genes found in the wet fementation metagenome (> 5 EGTs)** |
| --- | --- |
| CRISPR-associated protein GSU0053 (Cas_GSU0053) | Adenylate cyclase, class-I |
| Plant Basic Secretory Protein | Bacterial Na+/H+ antiporter B (NhaB) |
| Protein of unknown function (DUF3303) | Bacteriophage holin |
| Protein similar to CwfJ C-terminus 1 | C-terminal regulatory domain of Threonine dehydratase |
| Sterol-sensing domain of SREBP cleavage-activation | CRISPR-associated protein (Cas_Csy2) |
|  | Disulfide bond isomerase protein N-terminus |
|  | Domains of unknown function (DUF1987, DUF3492) |
|  | Glycosyltransferase family 10 (fucosyltransferase) |
|  | HSP20-like domain of unknown function (DUF1813) |
|  | Hint module |
|  | KR domain |
|  | Kinase binding protein CGI-121 |
|  | Leucine rich repeat variant |
|  | Mannosyl oligosaccharide glucosidase |
|  | Penicillin-insensitive murein endopeptidase |
|  | Peptidase C13 family |
|  | Plasmodium ookinete surface protein Pvs28 |
|  | Proteins of unknown function (DUF2983, DUF3737) |
|  | Replication protein A C terminal |
|  | Rib/alpha-like repeat |
|  | Spore coat associated protein JA (CotJA) |
|  | Transposase |
|  | tRNA methyltransferase complex GCD14 subunit |
|  | ribonuclease |
|  | Uncharacterised ACR, YagE family COG1723 |
|  | Uncharacterised protein families (UPF0158, UPF0164, DUF0227) |
|  | Uncharacterized protein domain (DUF2202) |
|  | Uncharacterized protein conserved in bacteria (DUF2062, DUF2090, DUF2186, DUF2262) |

Only EGTs featuring an abundance of 0 in the one and at least 5 in the other metagenome dataset were taken into account. Relative abundances were normalized based on the smallest dataset.
